# Supplementary material for: Mosquitoes of the Maculipennis complex in Northern Italy
Source: Sci Rep. 2021 Mar 19;11:6421. doi: 10.1038/s41598-021-85442-9 (PMC7979756; doi:10.1038/s41598-021-85442-9)

Mosquitoes of the Maculipennis complex in Northern Italy

Mattia Calzolari, Rosanna Desiato, Alessandro Albieri, Veronica Bellavia, Michela Bertola, Paolo Bonilauri, Emanuele Callegari, Sabrina Canziani, Davide Lelli, Andrea Mosca, Paolo Mulatti, Simone Peletto, Silvia Ravagnan, Paolo Roberto, Deborah Torri, Marco Pombi, Marco Di Luca, Fabrizio Montarsi

Supplementary information

Table S1. Mosquitoes of the Maculipennis complex identified at provincial level (NUT3).

	An. atroparvus	*An. maculipennis s.s.*	*An. melanoon*	*An. daciae sp. inq.*	Total
Veneto					
Belluno		2			2
Padova		48		40	88
Rovigo		34		283	317
Treviso		21		4	25
Venezia		41		80	121
Verona	10	15		296	321
Vicenza		6		5	11
Emilia-Romagna					
Bologna		5		26	31
Ferrara	1	10		287	298
Forli		6			6
Modena		28		52	80
Parma		13		3	16
Piacenza		3		11	14
Ravenna		1			1
Reggio E		19		29	48
Rimini		1			1
Friuli Venezia Giulia					
Gorizia		30		2	32
Pordenone		7		4	11
Udine		10		11	21
Lombardy					
Bergamo		14		4	18
Brescia		13		3	16
Cremona		15		12	27
Lodi		5	1	15	21
Mantova	17	45		180	242
Milano		21	3	83	107
Pavia		2	3	319	324
Varese		1		1	2
Piedmont					
Alessandria			6	42	48
Biella				2	2
Cuneo		1		1	2
Novara				71	71
Torino		1		5	6
Vercelli				160	160
Total	28	418	13	2031	2490

Table S2. Mosquito of the Maculipennis complex with reference to the year and method of sampling.

Year	Sampling method	*An. atroparvus*	*An. maculipennis s.s.*	*An. melanoon*	*An. daciae sp. inq.*	Total
2011	Trap		5		2	7
2013	Trap		10		38	48
2014	Trap		5		6	11
2015	Trap		14		19	33
2016	Trap				26	26
2017	Trap	8	103	4	618	733
	Aspiration	10	45		258	313
2018	Trap		103	7	617	727
	Aspiration	10	128	1	394	533
	Larval sampling		2		15	17
2019	Trap		3		39	42
Total		28	418	13	2031	2490

Table S3. Selected covariates after collinearity screening and their averaged contribution (Av.) to the obtained models and related confidence interval (CI).

		An. daciae sp. inq.		*An. maculipennis s.s.*	
Species	Code	Av.	CI	Av.	CI.
Precipitation of Wettest Quarter	bio16	0.4	0.6-0.2	0.3	0.5-0.1
Isothermality*	bio3	0.4	0.7-0.1	0.6	1.0-0.3
Min Temperature of Coldest Month	bio6	0.7	1.0-0.5	0.1	0.1-0.0
Temperature Annual Range	bio7	0.4	0.6-0.2	1	1.2-0.8
Corine Land Cover	clc2018	7	7.6-6.4	2.1	2.5-1.7
Middle Infra-red, amplitude of annual cycle	er1603a1	2.5	3.0-2.1	1.3	1.6-0.9
Middle Infra-red amplitude of tri-annual cycle	er1603a3	0.5	0.6-0.3	0.5	0.7-0.3

Middle Infra-red, proportion of total variance due to annual cycle	er1603d1	1.1	1.2-0.9	1.7	2.2-1.3
Middle Infra-red, proportion of total variance due to bi-annual cycle	er1603d2	1	1.1-0.8	3	3.5-2.5
Middle Infra-red, phase of tri-annual cycle	er1603p3	2.7	3.3-2.2	6.7	7.8-5.7
Day-time land surface temperature. amplitude of tri-annual cycle	er1607a3	1.4	2.1-0.8	1.2	1.6-0.8
Day-time land surface temperature, proportion of total variance due to tri-annual cycle	er1607d3	0.3	0.6-0.1	0.9	1.2-0.5
Day-time land surface temperature, % zero or above DN range	er1607e1	0.7	0.9-0.6	2.3	2.8-1.8
Day-time land surface temperature, total variance	er1607vr	1.5	2.0-1.0	0.1	0.2-0.1
Night-time land surface temperature, amplitude of bi-annual cycle	er1608a2	1.6	2.1-1.0	1	1.4-0.7
Night-time land surface temperature, amplitude of tri-annual cycle	er1608a3	1.3	1.8-0.8	1.1	1.4-0.9
Night-time land surface temperature, minimum value	er1608mn	0.6	0.8-0.3	0.2	0.4-0.1
Night-time land surface temperature, total variance	er1608vr	0.3	0.4-0.1	0.5	0.9-0.2
Enhanced vegetation index (EVI), fourier mean for entire time series	er1614a0	1.5	1.8-1.1	2.5	3.4-1.7
Enhanced vegetation index (EVI) Proportion of total variance due to annual cycle	er1614d1	1.4	1.7-1.1	1.9	2.4-1.4
Enhanced vegetation index (EVI),, total variance	er1614vr	3.7	4.3-3.2	1	1.8-0.2
Normalised difference vegetation index(NDVI) Amplitude of tri-annual cycle	er1615a3	1	1.2-0.8	1.4	1.7-1.0
Normalised difference vegetation index(NDVI), minimum value	er1615mn	2.2	2.8-1.6	6.2	7.1-5.3
Digital elevation model (altitude)	globedem	7.3	9.2-5.4	19.9	22.2-17.5
Digital elevation model (slope)	slope	8.2	9.7-6.7	7.3	9.2-5.3
Proximity raster of small inland bodies	inland_osm_proximity	8.8	9.4-8.2	5.9	6.9-4.9
Proximity raster of wetland	prox_clc4	6.9	8.3-5.5	5.7	6.7-4.7
Proximity raster of river	proxy_rivers	1.7	2.2-1.3	2.8	3.3-2.2
October precipitations	rain10	2.6	3.3-2.0	6.1	6.8-5.3
March precipitations	rain3	0.9	1.3-0.6	0.8	1.1-0.5
July precipitations	rain7	0.4	0.6-0.3	1.4	1.7-1.1
Proximity raster of ricefield	prox_ricefield	25.7	27.0-24.5	1.7	1.8-1.6
USDA soil classification	USDA Soil	2.6	2.9-2.3	9.5	12.1-6.9
Tree cover	tree_cp_2015	0.6	0.7-0.5	1.2	1.7-0.7

Figure S1. Points of presence of *An. melanoon* (a), *An. atroparvus* (b), *An. maculipennis s.s.* (c), *An. daciae sp.inq*. (d). Maps created using QGIS 3.10 (www.qgis.org); layers: Globe DEM (www.ngdc.noaa.gov), EU-Hydro (www.copernicus.eu).

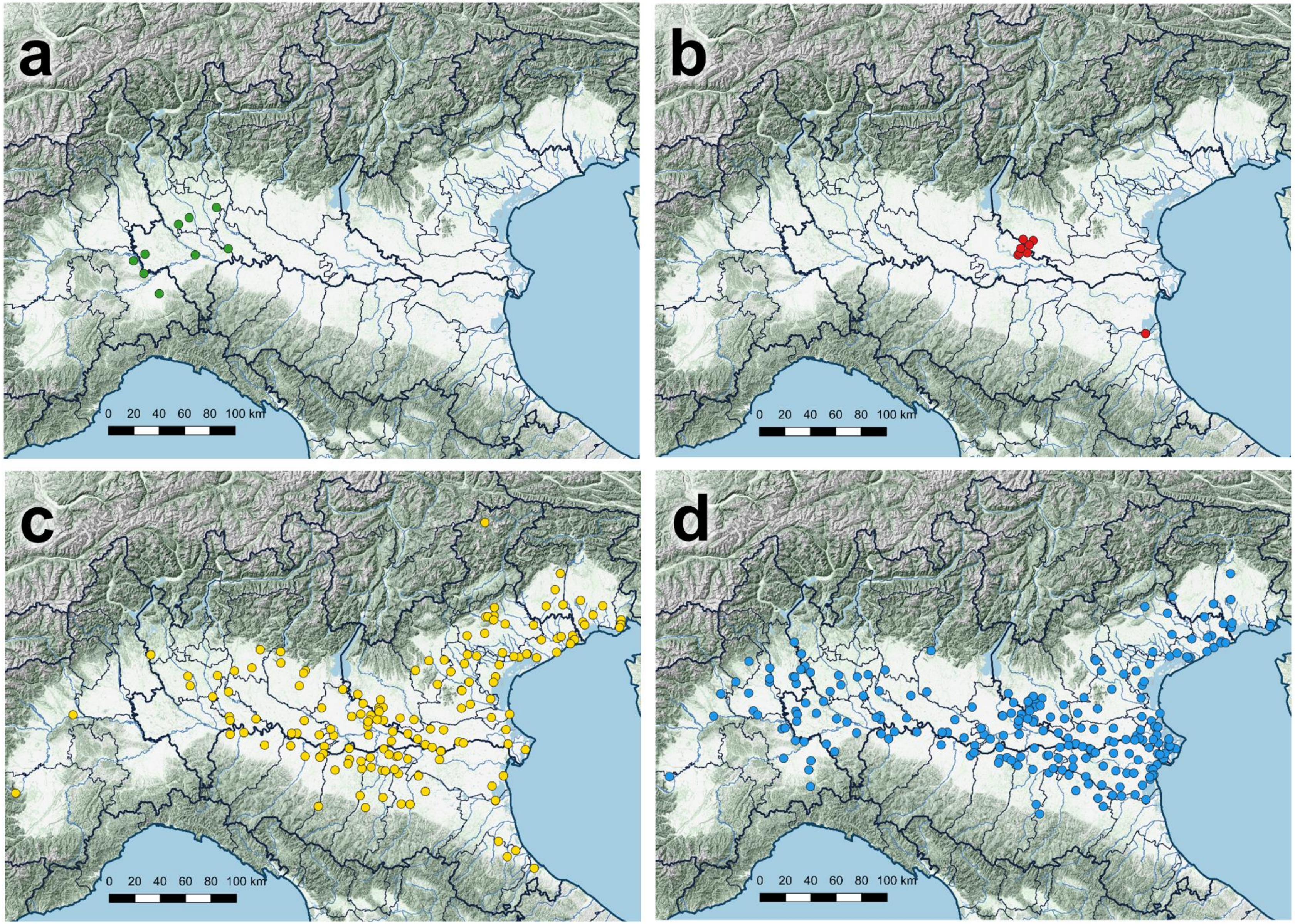

Figure S2. Examples of multiple peaks recorded in electropherograms.

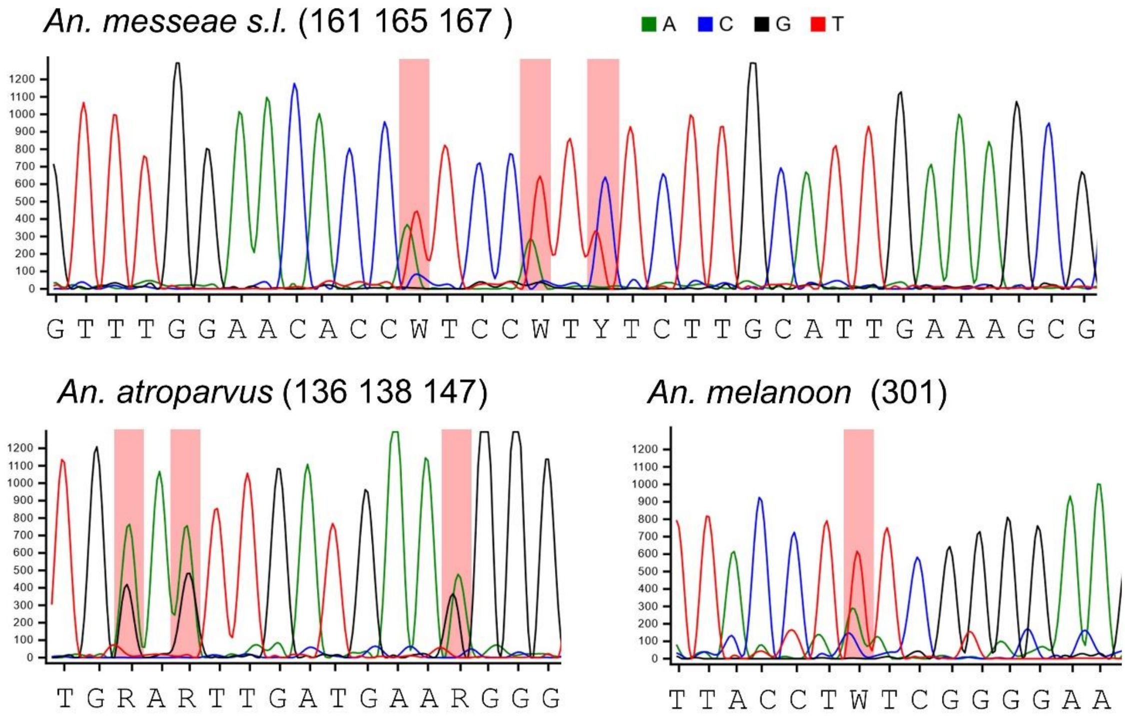

Graphic modified from https://www.gear-genomics.com/teal)

Figure S3 Relevance of soil classification in the ENM of *An. maculipennis s.s.* (white) with reference to the % area of different soil types in the surveyed area (grey).

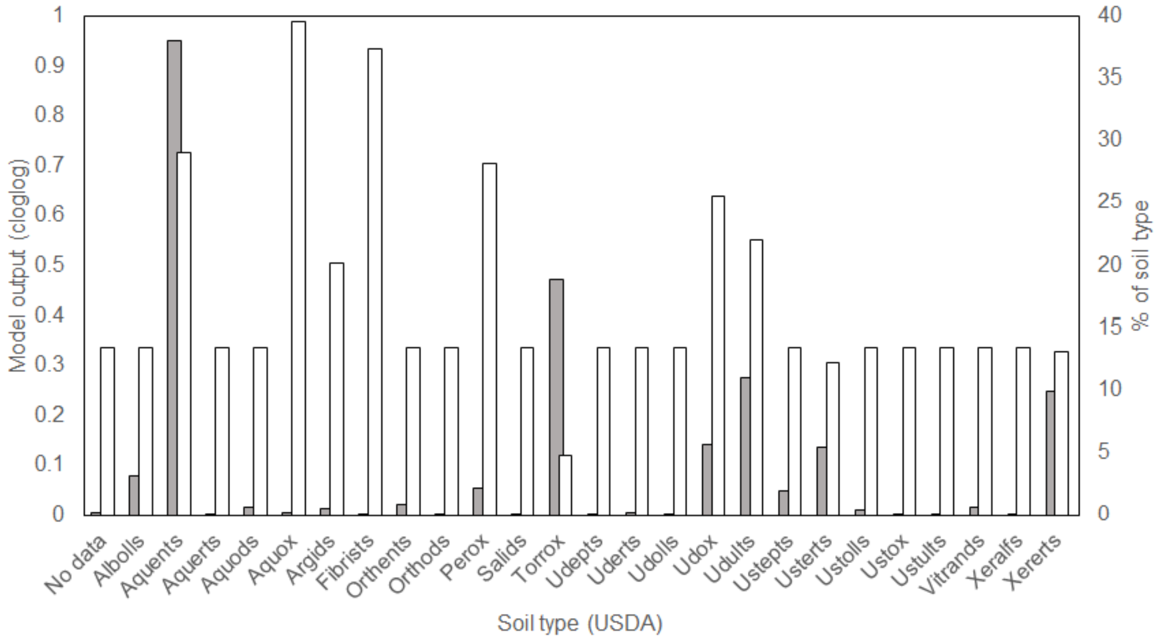

Figure S4. Response curve showing how the NDVI affects the two models constructed with this covariate. *An. daciae sp. inq.* in black, *An. maculipennis s.s.* in red.

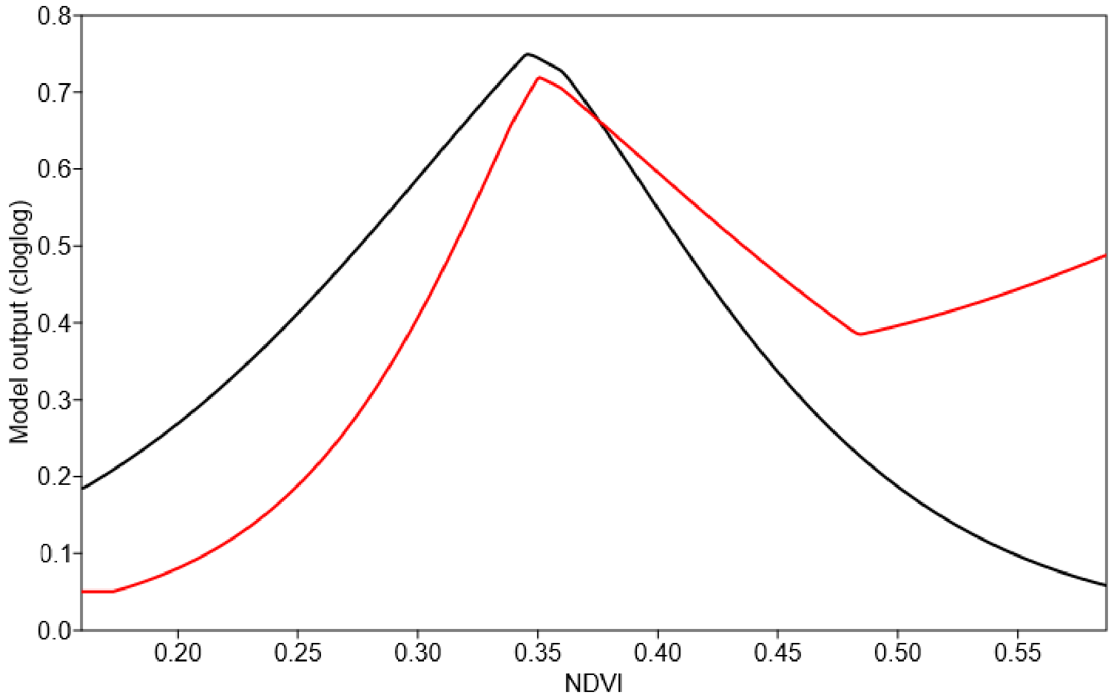

Figure S5. Response curve showing how the altitude affects the two models constructed with this covariate. *An. daciae sp. inq.* in black, *An. maculipennis s.s.* in red.

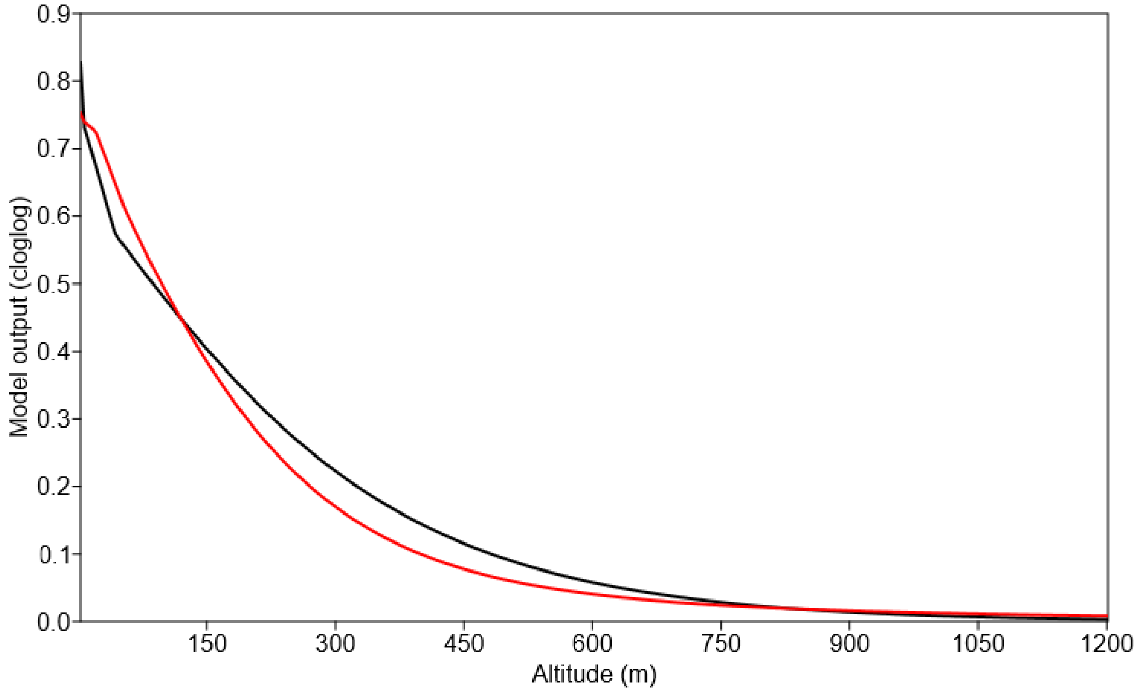

Supplement: Supplementary file 1 — Supplementary information. [file 41598_2021_85442_MOESM1_ESM.pdf]
